# Habitual Aerobic Exercise Is Associated with Reduced Negative Emotional Elicitation: An fNIRS Study

**DOI:** 10.3390/brainsci16040409

**Published:** 2026-04-11

**Authors:** Xuru Wang, Chenglin Zhou

**Affiliations:** 1 Shanghai Institute of Early Childhood Education, Shanghai Normal University, Shanghai 200234, China; rrwang@shnu.edu.cn; 2 School of Psychology, Shanghai University of Sport, Shanghai 200438, China

**Keywords:** habitual aerobic exercise, emotional elicitation, emotion regulation, cognitive reappraisal, expressive suppression, fNIRS, prefrontal cortex

## Abstract

**Objectives:** Habitual exercise has been associated with a lower risk of emotional disorders and greater emotional stability. However, it remains unclear whether the beneficial effects of habitual aerobic exercise primarily emerge during the stage of emotion elicitation or during the subsequent regulation of negative emotions. The present study examined the association of habitual aerobic exercise with the intensity of negative emotion elicitation, the effectiveness of emotion regulation strategies, and patterns of prefrontal activation measured using functional near-infrared spectroscopy. **Methods:** Sixty-four participants were recruited, including individuals with habitual aerobic exercise (trained group, *n* = 32) and those without regular exercise habits (untrained group, *n* = 32). Participants completed an emotion regulation task consisting of four conditions: viewing neutral images, viewing negative images, cognitive reappraisal, and expressive suppression. The comparison between neutral and negative viewing conditions was used to assess emotional elicitation, whereas comparisons between the regulation conditions and passive viewing of negative images were used to assess emotion regulation effects. **Results:** Compared with the untrained group, the trained group showed a lower emotional elicitation effect, accompanied by lower right ventrolateral prefrontal cortex (rVLPFC) activation during picture viewing at a more liberal threshold. The two groups showed similar reductions in negative emotional experience during emotion regulation, whereas individuals with habitual exercise showed a trend toward lower right dorsolateral prefrontal cortex (rDLPFC) activation during expressive suppression. **Conclusions:** Habitual aerobic exercise is associated with greater emotional stability, characterized by reduced initial emotional reactivity during emotion elicitation. Habitual exercisers may also show more efficient neural processing, reflected in distinct prefrontal activation patterns during emotional processing and regulation, although these exploratory findings require confirmation in future research.

## 1. Introduction

Emotions modulate subjective experience, action tendencies, and physiological responses, such as increased heart rate and heightened neural activity [1]. Emotional episodes have been implicated as triggering factors for cardiovascular events, and emotional dysfunction shows robust associations with the development of multiple diseases, including cancer [2,3]. Individuals differ in their emotional stability when facing adverse situations, with some—such as those carrying specific genetic variants—being more vulnerable to developing emotional symptoms [4]. Evidence from animal studies further suggests that long-term voluntary exercise can modulate epigenetic mechanisms, thereby promoting neural plasticity and reshaping the brain, particularly the prefrontal cortex [5]. Because life inevitably involves stressors and negative events, and most people experience at least one traumatic event, the ability to maintain emotional stability under such conditions represents a central component of physical and psychological well-being [6].

According to Gross’s two-stage dynamic process model, negative emotions are generated through bottom-up processing of emotional cues, whereby the evaluation of negative information engages limbic regions such as the amygdala and triggers emotional response tendencies, including subjective experience and expressive behavior [7]. These response tendencies can be actively regulated, allowing individuals to modulate the subsequent trajectory of their emotional reactions. Cognitive reappraisal and expressive suppression are two commonly used strategies for down-regulating ongoing emotional responses. Cognitive reappraisal involves construing a potentially emotion-eliciting situation in less affective terms (e.g., viewing a teacher’s criticism as constructive), whereas expressive suppression involves inhibiting emotion-expressive behaviors that are about to occur or are already ongoing (e.g., holding back tears when being criticized) [8]. Thus, emotional stability in the face of adversity may arise either from reduced emotional reactivity during the generation stage or from more efficient regulation of emotional responses once they have been elicited.

Recent research indicates that physical activity is associated with fewer negative emotional states and greater emotional stability in response to negative stimuli. Compared with physically inactive individuals, those who engage in regular exercise report fewer difficulties in emotion regulation and a lower risk of affective disorders [9,10]. These benefits appear to be particularly pronounced for aerobic exercise. Acute aerobic exercise has been shown to attenuate the intensity of state anxiety and negative affect elicited by aversive stimuli, and to reduce right-frontal EEG asymmetry, a neural marker associated with withdrawal-related and negative emotional states [11,12,13]. Long-term and habitual aerobic exercise has been shown to improve emotional well-being and general health, as well as to reduce overall mood disturbance [14,15,16]. Neurophysiological evidence further suggests that physically active individuals exhibit smaller event-related responses (e.g., N2 amplitudes) when viewing negative emotional images [17]. Although the emotion-stabilizing effects of aerobic exercise are well established, an important question concerns at which stage of emotional processing these effects emerge. Many prior studies have relied on questionnaires or passive viewing paradigms without specifying viewing or regulation strategies, leaving unresolved whether aerobic exercise primarily influences the generation of emotional responses or their subsequent regulation.

To address this question, a growing number of studies have examined how acute aerobic exercise modulates different stages of emotional processing, particularly the regulation stage. Acute bouts of aerobic exercise have been shown to increase oxygenated hemoglobin in prefrontal regions, and greater post-exercise activation of the left dorsolateral prefrontal cortex (DLPFC) is associated with better behavioral performance in cognitive reappraisal [18,19]. From a top–down perspective, prefrontal control regions modulate emotional responses by inhibiting bottom–up reactivity originating from subcortical structures such as the amygdala [20,21,22]. Consistent with this framework, acute aerobic exercise appears to transiently strengthen functional connectivity between prefrontal regions and the amygdala, a pathway that is central to effective emotion regulation [23]. However, these neuromodulatory effects are short-lived: as physiological arousal returns to baseline following exercise, the associated enhancements in regulatory capacity tend to diminish [24].

Importantly, the transient effects of single aerobic exercise sessions may accumulate over time through repeated engagement in aerobic activity, giving rise to more enduring functional and structural plasticity in the brain [25,26]. Habitual aerobic exercise is associated with sustained changes in neuromodulation and neural activity, which may consolidate with increasing training frequency and lead to homeostatic adaptations and long-term plastic changes [27,28]. Brain circuits implicated in both emotion generation and emotion regulation may therefore undergo gradual reorganization. In particular, the prefrontal cortex, which plays a central role in the flexible control of emotional responses and goal-directed behavior, shows structural sensitivity to long-term physical activity [29]. Greater prefrontal gray matter volume has been linked to better cognitive–emotional control, and regular aerobic exercise has been associated with increases in prefrontal gray matter volume [30,31]. These findings suggest that habitual aerobic exercise may be linked to a greater capacity of prefrontal systems to support emotional stability. Nevertheless, direct evidence remains limited regarding whether this link is primarily evident at the stage of emotion generation or during the subsequent regulation of negative emotions. Moreover, it remains unclear whether habitual exercisers and less active individuals show differential patterns of prefrontal activity across distinct stages of emotion processing. The present study aims to address these questions and clarify how habitual aerobic exercise shapes the neural dynamics underlying emotional stability.

## 2. Materials and Methods

### 2.1. Participants

Sixty-four university students were recruited from the same institution, including 32 trained participants with regular aerobic exercise habits (17 males) and 32 untrained participants without such habits (15 males). Exercise habits were primarily assessed by self-report. The trained group comprised students who had engaged in regular moderate- to high-intensity aerobic exercise at least four times per week, with each session lasting more than 45 min for at least 2 consecutive years. All trained participants were recruited from physical education or sports training majors, which required sport performance qualifications for admission, and their primary specializations included middle- and long-distance running, swimming, tennis, and badminton. By contrast, the untrained group comprised students from sport-related but non-athlete majors (e.g., sport rehabilitation and sport human sciences) who reported low physical activity levels (<30 min/day, <2 sessions/week) [32] and no regular exercise habits. All participants were aged 18–25 years, right-handed, reported no history of emotional disorders, had not taken psychotropic medication, and provided written informed consent after receiving a full explanation of the experimental procedures. Detailed demographic information for both groups is provided in Table 1. This study was approved by the local ethics committee (No. 102772019RT044).

### 2.2. Questionnaires

The International Physical Activity Questionnaire–Short Form (IPAQ-SF) was used to assess both physical activity levels, expressed in metabolic equivalent minutes (MET-min; higher scores indicate greater activity), as well as time spent sitting [33]. In addition, resting heart rate was measured using a Polar heart rate monitor (H10). The Beck Depression Inventory (BDI) was used to assess depressive symptoms [34], and the Trait version of the State–Trait Anxiety Inventory (STAI-T) was used to measure trait anxiety [35]. The Positive and Negative Affect Schedule (PANAS) was administered to evaluate affective well-being, including the two subscales of positive affect and negative affect [36]. The abbreviated Profile of Mood States (A-POMS) was used to assess seven dimensions of mood [37]. Total Mood Disturbance was calculated by subtracting the sum of the positive subscale scores from the sum of the negative subscale scores and adding a constant of 100, with higher scores indicating a more negative mood state. In addition, the Emotion Regulation Questionnaire (ERQ) was used to assess individuals’ habitual use of two emotion regulation strategies: cognitive reappraisal and expressive suppression [38]. These questionnaires were administered to characterize the sample.

### 2.3. Emotion Regulation Task

The task adopted a block-design picture-viewing paradigm consisting of four blocks, each representing one condition: viewing neutral pictures (WNeu), viewing negative pictures (WNeg), cognitive reappraisal (CR), and expressive suppression (ES). The WNeu block contained 30 neutral pictures and served as a non-emotional baseline, whereas the WNeg, CR, and ES blocks each used 30 negative pictures paired with distinct emotion regulation instructions. In WNeu and WNeg, participants passively viewed the pictures. In the CR condition, they reinterpreted the meaning of the pictures—for example, by questioning their realism or imagining more benign interpretations (e.g., seeing three large cockroaches as a “happy little family” and reminding themselves that every living creature has the right to exist). In the ES condition, participants inhibited outward emotional expressions and bodily reactions while continuing to view the pictures attentively, such that even an observer nearby would be unable to detect their internal emotional experience.

**Figure 1:**
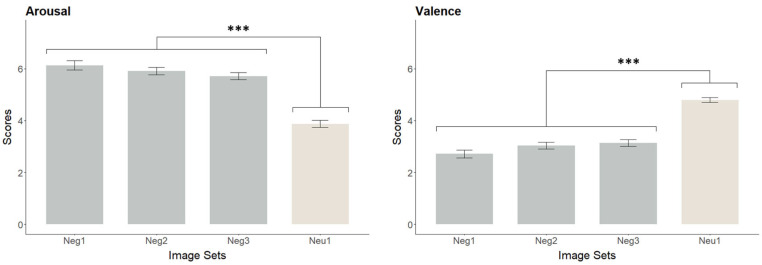
Comparison of arousal and valence rating results across image sets (Mean ± SE). *** *p* < 0.001.

A total of 120 pictures were selected from the International Affective Picture System (IAPS) [39], consisting of one set of neutral pictures for the WNeu condition and three sets of negative pictures (for the WNeg, CR, and ES conditions), with 30 pictures per set. The primary selection criterion was to ensure that the three negative sets were closely matched in valence and arousal ratings within the IAPS. Because cultural factors may influence emotional responses, the standardized affective norms of the IAPS may not fully apply to the present sample [40]. Therefore, an independent validation procedure was conducted to confirm that the three sets of negative pictures were equivalent in emotional attributes. Twenty additional raters (10 males, 10 females; M = 19.65 ± 1.60 years) who were blind to the study aims evaluated each picture’s valence and arousal using the standard 9-point IAPS Likert scales (valence: 1 = very unpleasant, 9 = very pleasant; arousal: 1 = very calm, 9 = very excited). One-way ANOVAs across the four picture sets revealed significant effects of both valence, *F*(3, 116) = 47.77, *p* < 0.001 and arousal, *F*(3, 116) = 52.45, *p* < 0.001. Bonferroni post hoc comparisons indicated that the neutral set differed significantly from all three negative sets (*p*s < 0.001), whereas no significant differences were found among the three negative sets (valence: *p*s > 0.13, arousal: *p*s > 0.30). These results confirmed that all negative picture sets effectively elicited negative affect and were comparable in their emotional properties (Figure 1, Appendix A Table A1).

**Figure 2:**
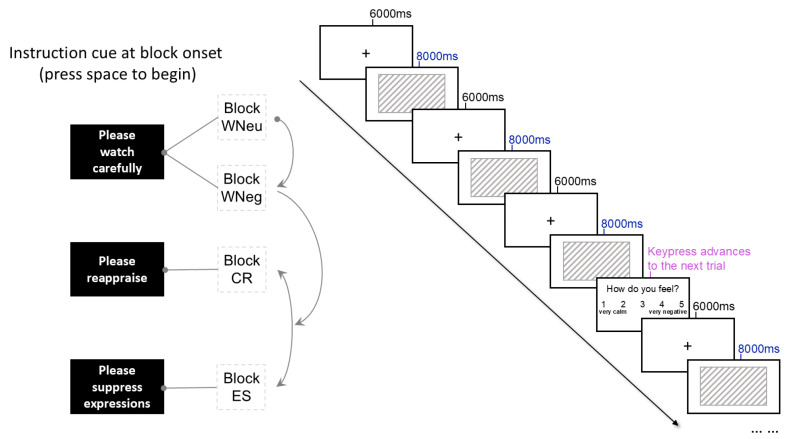
The flow chart of emotion regulation task.

Participants were seated in a quiet room approximately 100 cm from a 14-inch monitor. Before each condition, they were instructed on the task and completed six practice trials using pictures that did not appear in the formal experiment. At the beginning of each block, a brief cue (“Please watch carefully,” “Please reappraise,” or “Please suppress expressions”) was presented, and participants initiated the block by pressing the space bar. Each block consisted of 30 trials. Pictures were displayed for 8000 ms and separated by a 6000 ms inter-trial interval. After every third trial, participants rated their current emotional state on a 5-point Likert scale (1 = very calm, 5 = very negative) by pressing the corresponding number keys (Figure 2). The WNeu block was always presented first to avoid emotional carry-over effects, followed by the WNeg block [41]. The order of the CR and ES blocks was pseudo-randomized across participants. A two-minute break was provided between blocks to allow participants’ emotional states to return to baseline [40].

### 2.4. Picture Recall Task

The emotion regulation task lacked behavioral indicators (e.g., accuracy or reaction time) to verify participants’ engagement. To ensure that the observed effects were not confounded by inattention (e.g., looking away from the screen), a picture recall task was administered after the emotion regulation task. This task included both previously presented (“old”) and novel (“new”) pictures. The old pictures were drawn from those presented during the emotion regulation task, with 6 pictures selected from each of the 4 conditions (24 in total). The new pictures, which had not appeared previously, consisted of 18 negative and 6 neutral pictures that were matched to the old pictures in emotional valence and arousal (Appendix A Table A2).

The recall task comprised 48 trials in total. On each trial, one picture was presented, and participants indicated within 6000 ms whether the picture had appeared in the previous task by pressing the *Q* key (“appeared”) or the *P* key (“not appeared”). A fixation cross was then displayed for 500 ms before the next trial. Before the formal test, participants completed eight practice trials (four old and four new), and none of these pictures were included in the main task.

### 2.5. Functional Near-Infrared Spectroscopy (fNIRS)

**Figure 3:**
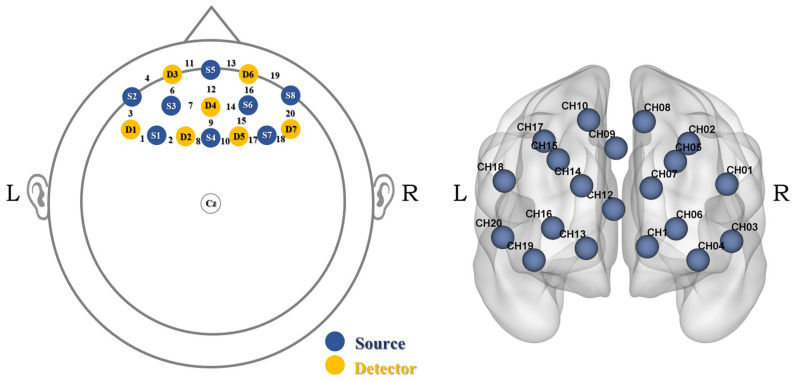
fNIRS probe placement. Sources S5 and S4 were positioned at FPz and Fz, and detector D4 at AFz (CH: channel).

fNIRS data were collected using a continuous-wave NIRSport2 system (NIRx)—NIRx Medical Technologies, LLC, Orlando, FL, USA. The system comprised 8 light sources and 7 detectors, forming 20 measurement channels with a source–detector separation of 3 cm over the prefrontal cortex (PFC) according to the international 10–20 EEG system (Figure 3). Channel–region correspondence was determined based on the previous literature [18,42,43]. The channels were categorized into six prefrontal subregions: the frontopolar prefrontal cortex (FPA; channels 6, 12, 16), the orbitofrontal cortex (OFC; channels 11, 13), the left ventrolateral prefrontal cortex (VLPFC; channels 1, 3, 4), the right VLPFC (channels 18–20), the left dorsolateral prefrontal cortex (DLPFC; channels 2, 5, 7, 8), and the right DLPFC (channels 10, 14, 15, 17). The FPA and OFC were treated as bilateral regions because they contained too few channels to distinguish between the two hemispheres. Each LED light source emitted light at two wavelengths (760 and 850 nm), and data were recorded at a sampling rate of 8.7 Hz using Aurora software (2023.9.6).

### 2.6. Procedures

Participants were recruited according to the predefined inclusion criteria for the trained and untrained groups. After providing written informed consent, they wore a heart rate monitor to measure resting heart rate and completed a series of questionnaires via a QR code, including the IPAQ-SF to assess physical activity levels, the BDI, STAI-T, PANAS, and ERQ. Subsequently, they completed the emotion regulation task and picture recall task in this fixed order, while fNIRS signals were recorded over the PFC during the former. To reduce deliberate memorization of the stimuli, participants were informed during the written consent procedure only that a memory test would be administered and that it might include potentially unpleasant images.

### 2.7. Statistical Methods

#### 2.7.1. Emotion Regulation Task

**Behavioral Data** The task included two components: ***the emotion elicitation effect*** and ***the emotion regulation effect***. The emotion elicitation effect was examined by using trials with neutral images as the baseline and comparing subjective emotional experience during passive viewing of negative versus neutral pictures. The emotion regulation effect was examined by using passive viewing of negative images as the baseline and comparing subjective emotional experience during the use of regulation strategies (cognitive reappraisal and expressive suppression) relative to passive viewing. To investigate ***the emotion elicitation effect***, we conducted a repeated-measures ANOVA with condition (stimulus type: WNeu vs. WNeg) as the within-subject factor and group (trained vs. untrained) as the between-subject factor. By testing the main effect of stimulus type and its interaction with group, we assessed whether the negative pictures successfully induced negative emotional responses and whether this elicitation process was influenced by individuals’ habitual exercise levels.

We then examined ***the emotion regulation effect*** using a repeated-measures ANOVA with condition (viewing strategy: WNeg, CR/ES) as the within-subject factor and group as the between-subject factor. If a significant main effect of regulation strategy was observed, we compared CR and ES against WNeg to determine whether cognitive reappraisal and expressive suppression effectively reduced negative emotional experience. We further examined the strategy × group interaction to test whether the magnitude of regulation differed as a function of exercise habits.

To reduce the influence of extreme values, we excluded trials with reaction times shorter than 150 ms, as well as trials exceeding ±3 standard deviations from the individual mean.

**fNIRS Data** Raw optical intensity signals were preprocessed using the Homer2 toolbox (MGH–Martinos Center for Biomedical Imaging, Boston, MA, USA) running in MATLAB 2013b (MathWorks, Natick, MA, USA). As an initial preprocessing step, channels with extreme intensity values or a signal-to-noise ratio (SNR) below 2 were excluded, after which the remaining signals were transformed into optical density. Artifact detection was performed on 1.5 s segments; when signal fluctuations exceeded 5 SDs (the standard deviation threshold) or 50 times the signal amplitude (the amplitude threshold), a 2 s window surrounding the artifact was identified for correction. Motion artifacts were corrected using cubic spline interpolation [44]. Artifact detection was subsequently repeated using the same criteria and procedure, and trials containing artifacts that could not be successfully corrected were excluded from further analysis. Low- and high-pass filtering were not performed, as polynomial drift correction was incorporated into the later general linear model (GLM) analyses. These signals were then converted into changes in oxyhemoglobin (HbO), deoxyhemoglobin (HbR), and total hemoglobin (HbT) based on the modified Beer–Lambert law with wavelength-specific partial pathlength factors (PPF = 6.0) [45]. Guided by neurovascular coupling theory—according to which neural activation increases cerebral blood flow, resulting in elevated HbO and decreased HbR—we focused primarily on HbO signals, given their higher sensitivity to task-evoked activity [46,47,48].

The preprocessed HbO time series were analyzed using a GLM implemented in SPM8 (Wellcome Department of Cognitive Neurology) and NIRS-SPM v4 (Bio-Imaging Signal Processing Lab). A wavelet-MDL detrending algorithm and an HRF low-pass filter were applied to attenuate low-frequency drift, Mayer waves, respiration, and cardiac artifacts, ensuring that the signals primarily reflected cerebral hemodynamic responses. Task regressors were constructed based on the onset and duration of picture presentation, and GLM estimation produced *β*-coefficients for each channel and each condition. For further statistical analyses, *β*-values were averaged across channels belonging to the six predefined prefrontal ROIs to extract region-level activation indices.

The statistical approach used for the fNIRS-based *β*-values mirrored that of the behavioral data. For the ***emotion elicitation*** analysis, a repeated-measures ANOVA was conducted with stimulus type (WNeu vs. WNeg) and group as factors. For the ***emotion regulation*** analysis, a repeated-measures ANOVA was conducted using viewing strategy (WNeg, CR/ES) and group. As these ANOVAs were conducted separately for each of the six prefrontal ROIs, multiple-comparison correction was performed at the ROI level. Specifically, false discovery rate (FDR) correction was applied separately to the main effects and interaction effects obtained from the emotion elicitation, cognitive reappraisal, and expressive suppression analyses.

When the assumption of sphericity was violated, Greenhouse–Geisser corrected degrees of freedom (rounded to integers) were applied. Post hoc pairwise comparisons were adjusted using the Bonferroni–Holm method. Effect sizes for ANOVA results were reported as partial eta squared ($\eta_{p}^{2}$). All statistical analyses were carried out in R (version 4.5.1) using the bruceR package [49]. Effect sizes were reported as partial eta squared ($\eta_{p}^{2}$) for ANOVAs and Cohen’s *d* for *t*-tests.

#### 2.7.2. Picture Recall Task

Trials with reaction times shorter than 150 ms were excluded. Recognition accuracy, reaction time, and correct-trial reaction time were computed, and group differences were assessed using independent-samples *t*-tests. These comparisons were conducted to verify that any observed group differences in the emotion regulation task were not attributable to differences in attentional engagement during picture viewing. Participants with recognition accuracy below the passing criterion of 60% were considered not to have adequately completed the emotion regulation task adequately and were excluded from all behavioral and fNIRS analyses.

## 3. Results

### 3.1. Behavioral Results

#### 3.1.1. Picture Recall Task

**Figure 4:**
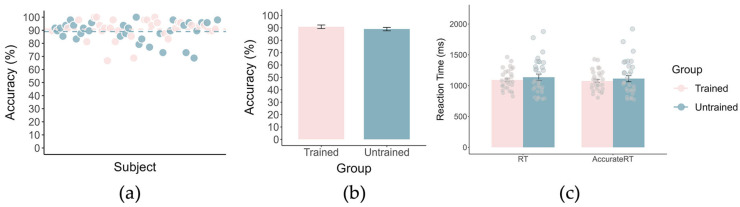
Results of the picture recall task. (**a**,**b**) Accuracy. (**c**) Reaction Time. Pink represents the trained group, and blue represents the untrained group. The dashed lines indicate the mean values.

All participants achieved accuracy levels above the 60% criterion; therefore, no participants were excluded due to inadequate engagement in the emotion regulation task. Independent-samples *t*-tests on recognition accuracy, reaction time, and correct-trial reaction time revealed no significant group differences (all *p*s > 0.36), indicating comparable task engagement between the trained and untrained groups (Figure 4).

#### 3.1.2. Emotion Regulation Task

**Figure 5:**
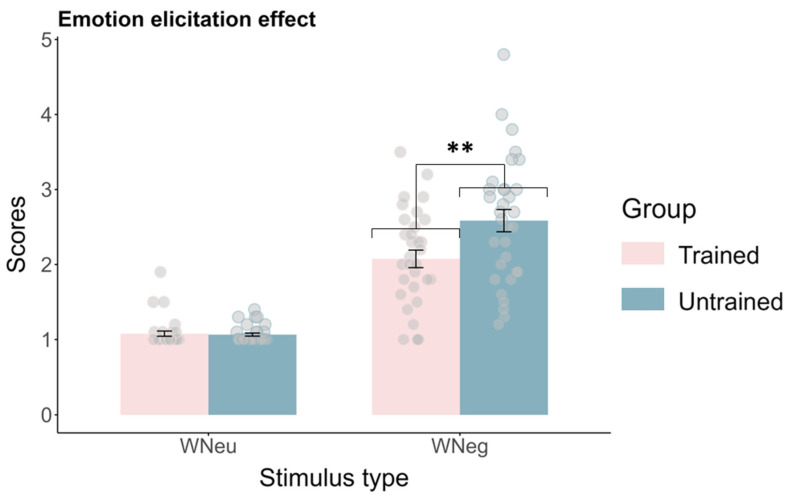
Emotion elicitation effect in the emotion regulation task (Mean ± SE). ** *p* < 0.01.

***Emotion elicitation effect***. A 2 (stimulus type: WNeu vs. WNeg) × 2 (group: trained vs. untrained) ANOVA revealed significant main effects of stimulus type (*F*(1, 62) = 194.01, *p* < 0.001, $\eta_{p}^{2}$ = 0.76) and group (*F*(1, 62) = 5.90, *p* = 0.018, $\eta_{p}^{2}$ = 0.09), as well as a significant stimulus type × group interaction (*F*(1, 62) = 8.35, *p* = 0.005, $\eta_{p}^{2}$ = 0.12). Follow-up simple-effects analyses showed that, in both the trained and untrained groups, emotional ratings were significantly higher in the WNeg condition than in the WNeu condition (*p*s < 0.001), confirming that the negative images effectively elicited negative emotional responses in both groups. In the WNeu condition, no significant difference in emotional ratings was observed between groups (*p* = 0.76). However, in the WNeg condition, the untrained group (2.58 ± 0.13) reported significantly higher emotional ratings than the trained group (2.08 ± 0.13), *t*(62) = 2.70, *p* = 0.009, Cohen’s *d* = 0.71, indicating a stronger negative emotional response among individuals without regular aerobic exercise habits (Figure 5).

**Figure 6:**
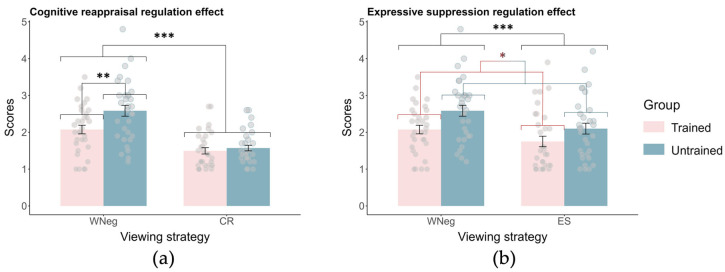
Emotion regulation effect in the emotion regulation task (Mean ± SE). (**a**) Cognitive reappraisal. (**b**) Expressive suppression. * *p* < 0.05, ** *p* < 0.01, *** *p* < 0.001.

***Emotion regulation effect***. A 2 (viewing strategy: WNeg vs. CR) × 2 (group: trained vs. untrained) ANOVA revealed significant main effects of viewing strategy (*F*(1, 62) = 97.85, *p* < 0.001, $\eta_{p}^{2}$ = 0.61) and group (*F*(1, 62) = 4.75, *p* = 0.033, $\eta_{p}^{2}$ = 0.07), as well as a significant viewing strategy × group interaction (*F*(1, 62) = 7.30, *p* = 0.009, $\eta_{p}^{2}$ = 0.11). Follow-up simple effects analyses of the interaction revealed that, in both the trained and untrained groups, emotional ratings were significantly higher in the WNeg condition than in the CR condition (*p*s < 0.001), confirming that cognitive reappraisal effectively reduced negative emotional experience across both groups. Notably, despite lower emotional ratings in the trained group under the WNeg condition (as described above), no significant group difference was observed in the CR condition (*p* = 0.52) (Figure 6a).

A 2 (viewing strategy: WNeg vs. ES) × 2 (group: trained vs. untrained) ANOVA revealed significant main effects of viewing strategy (*F*(1, 62) = 33.70, *p* < 0.001, $\eta_{p}^{2}$ = 0.35) and group (*F*(1, 62) = 5.41, *p* = 0.023, $\eta_{p}^{2}$ = 0.08). The viewing strategy × group interaction was not significant (*p* = 0.26). Follow-up comparisons indicated that emotional ratings were significantly higher in the WNeg condition (2.33 ± 0.09) than in the ES condition (1.93 ± 0.10), and that the trained group (1.91 ± 0.13) reported significantly lower emotional ratings than the untrained group (2.34 ± 0.13) (Figure 6b).

Given the significant between-group difference in PANAS negative affect scores, baseline PANAS negative affect was included as a covariate. The pattern of behavioral results related to emotion elicitation and emotion regulation remained unchanged after controlling for this covariate (see Appendix A Table A3 for the statistical results before and after covariate adjustment).

### 3.2. fNIRS Results

Data from four participants were excluded due to technical issues, including trigger signal loss and movement-related cable disruption during the presentation of negative images. An additional two participants were excluded due to low signal quality characterized by a poor signal-to-noise ratio. As a result, data from 58 participants (trained: *n* = 28; untrained: *n* = 30) were included in the final analyses. After applying the more stringent ROI-level correction for multiple comparisons, none of the effects that had reached significance before correction remained significant. The following results based on the more liberal uncorrected threshold are therefore reported for exploratory purposes.

**Figure 7:**
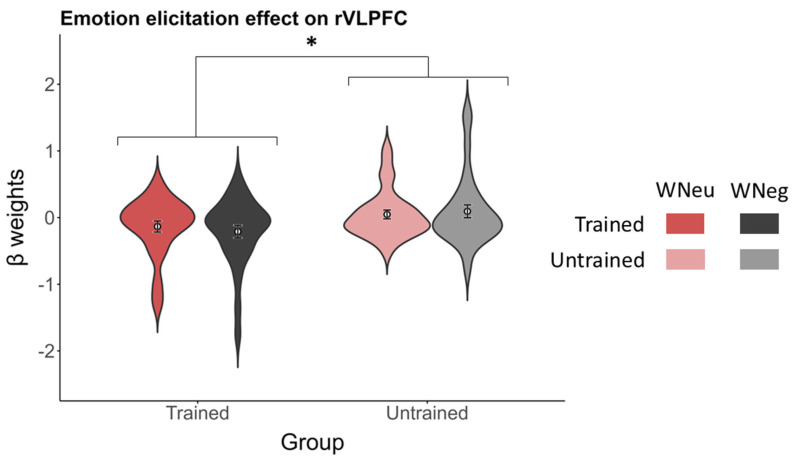
The emotion elicitation effect on rVLPFC. Red and gray bars represent the WNeu and WNeg conditions, respectively. Darker shades denote the trained group, whereas lighter shades denote the untrained group. * *p* < 0.05.

***Emotion elicitation effect***. For each of the six predefined PFC ROIs, a 2 (stimulus type: WNeu vs. WNeg) × 2 (group: trained vs. untrained) ANOVA was conducted. A main effect of group was observed in the rVLPFC at the more liberal threshold, *F*(1, 56) = 4.49, *p* = 0.039, $\eta_{p}^{2}$ = 0.07, with higher activation in the untrained group (0.07 ± 0.08) than in the trained group (−0.17 ± 0.08) (Figure 7). The main effect of stimulus type (*p* = 0.71) and the stimulus type × group interaction (*p* = 0.13) were not significant. None of the remaining ROIs showed significant effects (all *p*s > 0.12).

**Figure 8:**
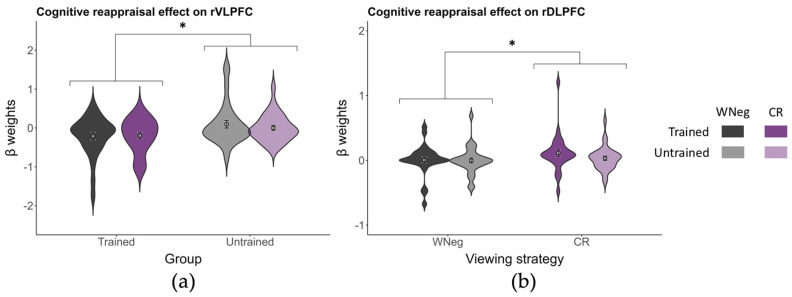
The cognitive reappraisal regulation effect on (**a**) rVLPFC and (**b**) rDLPFC. Gray and purple bars represent the WNeg and CR conditions, respectively. Darker shades denote the trained group, whereas lighter shades denote the untrained group. * *p* < 0.05.

***Emotion regulation effect***. Using the same ROI-based approach, a 2 (viewing strategy: WNeg vs. CR) × 2 (group: trained vs. untrained) ANOVA was conducted. At the more liberal threshold, the rVLPFC showed a group effect, *F*(1, 56) = 5.11, *p* = 0.028, $\eta_{p}^{2}$ = 0.08, with higher activation in the untrained group (0.05 ± 0.08) than in the trained group (−0.21 ± 0.08), whereas neither the main effect of viewing strategy (*p* = 0.38) nor the viewing strategy × group interaction (*p* = 0.27) was significant (Figure 8a). In the rDLPFC, a viewing strategy effect was observed in the uncorrected analysis, *F*(1, 56) = 5.05, *p* = 0.029, $\eta_{p}^{2}$ = 0.08, with higher activation in the CR condition (0.07 ± 0.03) than in the WNeg condition (0.00 ± 0.03) (Figure 8b). The main effect of group (*p* = 0.40) and the viewing strategy × group interaction (*p* = 0.27) were not significant. No significant main effects or interactions were detected in the remaining ROIs (all *p*s > 0.10).

**Figure 9:**
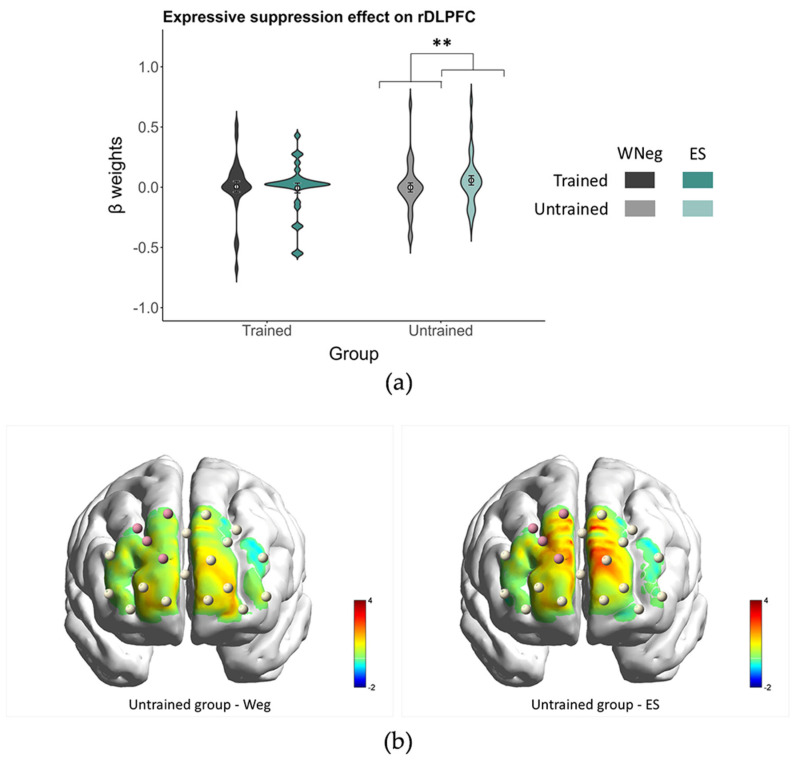
The expressive suppression regulation effect on rDLPFC. (**a**) Interaction effect of viewing strategy × group on rDLPFC activation. Gray and green bars represent the WNeg and CR conditions, respectively. Darker shades denote the trained group, whereas lighter shades denote the untrained group. ** *p* < 0.01. (**b**) Rendering of PFC activation. Colors indicate *t*-values of mean [HbO] relative to zero. Pink spheres denote channels in the rDLPFC showing significant differences. Brain renderings were generated using BrainNet Viewer [50].

A similar ANOVA was conducted with viewing strategy (WNeg vs. ES) and group. The viewing strategy × group interaction showed a significant effect in the rDLPFC in the uncorrected analysis, *F*(1, 56) = 4.26, *p* = 0.044, $\eta_{p}^{2}$ = 0.07, whereas the main effects of viewing strategy (*p* = 0.17) and group (*p* = 0.60) were not significant. Follow-up simple effects analyses indicated that, in the untrained group, activation in the rDLPFC was higher in the ES condition (0.06 ± 0.04) than in the WNeg condition (0.00 ± 0.04), *t*(56) = 2.50, *p* = 0.016, Cohen’s *d* = 0.46. In contrast, no significant difference between ES and WNeg conditions was observed in the trained group (*p* = 0.65) (Figure 9). None of the remaining ROIs showed significant effects (all *p*s > 0.05).

Baseline PANAS negative affect was included as a covariate in the analyses of the neural findings that had shown significant effects (Appendix A Table A4). The neural effects related to emotion elicitation remained significant after covariate adjustment. In contrast, the main effect of viewing strategy in the rDLPFC during cognitive reappraisal (i.e., greater activation during reappraisal than during passive viewing of negative stimuli) was no longer significant after controlling for this covariate, suggesting that this activation effect was sensitive to model adjustment and showed limited robustness.

### 3.3. Correlation Analyses

Given the significant viewing strategy × group interaction observed in the rDLPFC, we further examined whether expressive suppression–related neural changes were associated with behavioral regulation effects. Difference scores were computed for emotional ratings (WNeg − ES) and rDLPFC HbO *β*-values (WNeg − ES). Correlation analyses revealed no evidence of an association between expressive suppression–related changes in rDLPFC HbO *β*-values and reductions in negative emotional ratings in either group (untrained: *p* = 0.31; trained: *p* = 0.60).

## 4. Discussion

The present study used fNIRS to examine whether habitual aerobic exercise is associated with emotional stability when individuals encounter negative situations, and whether this association is evident at the stage of emotion elicitation and/or during subsequent emotion regulation. The results indicate that the advantage associated with habitual aerobic exercise primarily emerged at the stage of emotional elicitation. Habitual exercisers reported lower negative emotional experience when exposed to negative stimuli without explicit regulation. This attenuation was not accompanied by increased prefrontal activation, indicating that it did not rely on greater cognitive effort or regulatory load. In contrast, the present study did not observe a significant association between habitual aerobic exercise and the regulatory effectiveness of cognitive reappraisal or expressive suppression. However, individuals with habitual aerobic exercise tended to show reduced prefrontal activation despite achieving comparable regulatory outcomes.

### 4.1. Emotion Elicitation

Exposure to negative images elicited stronger subjective negative affect than neutral images in both groups, indicating that the emotional stimuli were effective in inducing negative emotional responses. This emotional reactivity was modulated by habitual exercise. Participants with regular exercise habits reported lower levels of negative emotional experience when viewing negative images compared with those without such habits. This pattern is consistent with the demographic results obtained from the PANAS questionnaire, which also showed lower negative affect scores among habitual exercisers. Notably, several studies examining acute aerobic exercise interventions have not observed significant changes in emotional reactivity to negative stimuli. For instance, research using a 30 min bout of moderate-intensity aerobic exercise found no difference in subjective emotional responses to negative pictures before and after exercise [51]. Similarly, electrophysiological studies have reported no significant changes in the LPP, a neural marker of emotional arousal, when participants viewed negative stimuli before and after interval aerobic exercise [52]. These findings suggest that the reduced emotional reactivity observed in the present study may reflect a trait-like effect associated with long-term aerobic exercise engagement rather than the transient effects of acute physical activity. Furthermore, this association does not appear to depend strongly on exercise intensity. Evidence suggests that habitual physical activity, regardless of whether it is of low, moderate, or high volume, is associated with a lower risk of emotional problems compared with a sedentary lifestyle [53].

Habitual exercisers showed a trend toward lower activation in the rVLPFC when viewing both neutral and negative images. This finding is consistent with the results reported by Giles, who observed reduced overall prefrontal activation in physically active individuals during exposure to emotional images [54]. One possible explanation is that long-term exercise may promote functional plasticity in the prefrontal cortex, potentially contributing to greater neural efficiency during cognitive and emotional processing. In this context, neural efficiency refers to reduced neural resource consumption while achieving comparable behavioral outcomes [55,56]. The VLPFC is commonly regarded as an appraisal-related region that is engaged during emotion generation and further recruited during cognitive reappraisal [57]. When individuals view emotional images, some degree of appraisal or interpretation inevitably occurs, regardless of the valence of the stimulus (e.g., whether the image depicts a table or a crying face). Because the difference in rVLPFC activation appeared only as a trend at the between-group level, rather than as a condition-specific effect, caution is warranted in interpreting this finding. Another possible explanation is that long-term exercise habits may be associated with alterations in cerebrovascular regulatory characteristics, such as arterial compliance and cerebrovascular reactivity [58]. As a result, when neural activity is elicited in the rVLPFC during picture viewing, the corresponding hemodynamic response pattern may differ between habitual exercisers and non-exercisers, partly reflecting variations in vascular properties. In addition, this finding may also be related to differences in viewing strategies adopted by the two groups during picture viewing. Previous studies have shown that even during passive viewing, participants may spontaneously engage in internal processing, such as differing degrees of psychological distancing from or immersion in the picture content, which may in turn contribute to differences in prefrontal activation [59].

### 4.2. Emotion Regulation

#### 4.2.1. Cognitive Reappraisal

Compared with passive viewing of negative images, cognitive reappraisal significantly reduced participants’ subjective negative affect in both groups, consistent with previous findings demonstrating the regulatory efficacy of this strategy [60]. The fNIRS results suggested a trend toward greater activation in the rDLPFC during cognitive reappraisal than during passive viewing of negative stimuli. The DLPFC has been consistently implicated in cognitive reappraisal processes [61], particularly the anterior portion, which has been linked more closely to emotion regulation than to the generation of negative emotion [57]. Reappraisal has been linked to top-down control exerted by the rDLPFC over attention-related regions [62]. Supporting this account, direct epidural stimulation of the DLPFC has been shown to reduce visual attention to emotionally salient stimuli [63]. However, the present data did not reveal a significant difference in rDLPFC activation during reappraisal between individuals with and without exercise habits. This result is consistent with the results reported by Giles [54], suggesting that the recruitment of this region during cognitive reappraisal may be independent of habitual physical activity. Notably, this condition effect in the rDLPFC was no longer significant after controlling for baseline PANAS negative affect. Although the covariate itself was not significant, this result suggests that the observed effect was sensitive to adjustment for negative affect experienced over the past week.

The present study did not observe greater regulatory benefits of cognitive reappraisal in habitual exercisers relative to individuals without aerobic exercise habits. Despite showing lower emotional reactivity during passive viewing of negative images, their subjective emotional ratings after applying the reappraisal strategy were comparable to those of participants without exercise habits. One possible explanation is that participants with exercise habits may already appraise negative stimuli in a relatively more balanced manner. Previous research suggests that physically active individuals may be more inclined to adopt more positive perspectives when interpreting negative situations [64], potentially limiting the additional benefit of explicit cognitive reappraisal in the present paradigm. Interestingly, Giles reported that improvements in reappraisal effectiveness increased with higher levels of physical activity [54]. Both the present study and the work by Giles used IAPS images as stimuli with comparable levels of negativity. However, in the present study, the mean emotional ratings during reappraisal were close to the lower bound of the scale, and the ratings obtained during passive viewing were also lower than those reported by Giles. This discrepancy may reflect differences in sex composition, with Giles’s work including a higher proportion of female participants and our study including equal numbers of males and females. Because females tend to be more sensitive to negative emotional stimuli, samples with more female participants may exhibit higher baseline negative affect [65,66], thereby providing greater scope for emotional reduction during reappraisal, as in Giles’s study.

Taken together, two interpretations may explain the absence of exercise-related advantages in reappraisal outcomes. First, habitual aerobic exercise may not substantially influence the effectiveness of cognitive reappraisal. Because habitual exercisers experience lower baseline emotional reactivity, they tend to encounter fewer situations in daily life that require deliberate emotion regulation. Second, the lower level of negative emotional arousal observed in the trained group may have limited the potential for further emotional reduction through reappraisal. This raises the possibility that individuals with habitual aerobic exercise possess an advantage in cognitive reappraisal, but the level of negative emotional arousal elicited by the experimental materials may have been insufficient to reveal this advantage. Future studies could clarify this possibility by using more highly arousing and negatively valenced stimuli to elicit stronger emotional responses and allow greater opportunity for emotion regulation.

#### 4.2.2. Expressive Suppression

The present findings showed that expressive suppression significantly modulated participants’ subjective emotional experience, consistent with previous studies [51,67]. By inhibiting facial and bodily expressions, this strategy may shift attentional focus away from internal negative emotional experiences toward the monitoring and control of one’s own expressive behaviors [68].

The magnitude of emotional improvement produced by expressive suppression did not differ significantly between the two groups. However, habitual exercisers reported lower levels of negative emotional experience after applying the suppression strategy. Because expressive suppression regulates emotions primarily by controlling outward expression rather than by altering the interpretation of emotional stimuli [38], this pattern may reflect differences in baseline emotional reactivity. Habitual exercisers tend to show less negative initial responses to negative stimuli, which may result in lower subjective negative emotional experience following suppression.

As an exploratory finding, only the untrained group showed increased rDLPFC activation during expressive suppression relative to passive viewing. The DLPFC has been identified as a neural substrate for the implementation of cognitive control [69]. Previous research has shown that the rDLPFC is involved in expressive suppression [70,71]. This region may support the maintenance of regulatory goals and strategies in working memory [72], thereby facilitating top-down control under emotionally conflicting conditions and contributing to response selection processes [73]. In the present study, the absence of increased rDLPFC activation in habitual exercisers may be tentatively interpreted as reflecting greater neural efficiency during expressive suppression. One possible explanation is that long-term aerobic exercise may be associated with functional adaptation in the prefrontal cortex [18,20,74], enabling expressive suppression to be implemented with fewer prefrontal resources. However, correlational analyses did not reveal a significant association between rDLPFC activation and the regulatory effectiveness of expressive suppression in either group. Therefore, the interpretation in terms of neural efficiency should not be considered definitive. Alternatively, neural efficiency in the DLPFC may be reflected in more efficient information transfer to deeper subcortical regions involved in emotion generation [26]. The fNIRS technique used in the present study does not allow this possibility to be directly examined. Future studies using fMRI may help clarify this issue. In addition, our inference was based primarily on participants’ self-reported emotional experience, and we did not directly record facial expressions or body posture during the task. Therefore, we cannot determine whether the two groups achieved comparable effects on the behavioral control of emotional expression. Moreover, lower rDLPFC activation may also reflect between-group differences in task engagement, effort allocation, or the intensity with which the suppression strategy was implemented. Given that the behavioral results showed lower initial reactivity to negative stimuli in habitual exercisers, they may have required fewer cognitive control resources during expressive suppression.

## 5. Conclusions and Limitations

Habitual aerobic exercise is linked to the early stage of emotion elicitation and to greater emotional stability. However, neural regions involved in emotion elicitation also include deeper structures such as the amygdala, cingulate cortex, and insula, which cannot be assessed with the present fNIRS approach. Future research using fMRI could further investigate the neural mechanisms underlying this effect, particularly by examining subcortical regions involved in emotion generation, such as the amygdala.

The present study did not find evidence for an association between habitual aerobic exercise and the effectiveness of cognitive reappraisal. However, habitual exercisers interpret negative stimuli in a less negative manner, potentially limiting the scope for further emotional regulation. In other words, habitual exercise may indeed be associated with emotion regulation capacity, but this association may not have been observed in the present study because the experimental stimuli may not have been sufficiently high in arousal or negative in valence. This possibility could be further examined in future research by employing more strongly negative emotional stimuli.

Individuals with habitual exercise showed a tendency toward lower prefrontal activation during both emotion elicitation and emotion regulation. Although this pattern was not robust in the present study and should therefore be regarded as exploratory, it may tentatively reflect greater neural efficiency. At the same time, alternative explanations remain possible, including differences in picture-viewing or emotion-regulation strategies, as well as physiological characteristics such as cerebrovascular regulation. Future research could help clarify this issue by incorporating resting prefrontal activity as a covariate and by more directly assessing individual differences in strategy use.

Several other limitations of this study should also be noted. Because the present findings are based on cross-sectional group comparisons, causal conclusions regarding the effects of habitual aerobic exercise on emotional reactivity and regulation cannot be drawn. Future research employing long-term exercise interventions is needed to provide stronger causal evidence. In addition, the emotion regulation task followed a fixed block order, with neutral viewing presented first, followed by negative viewing, and then cognitive reappraisal and expressive suppression in a pseudorandomized between-subjects order. This design was intended to reduce carryover effects across conditions, but it may also have introduced order-related influences that affected responses in the later regulation blocks. Future studies could address this issue by separating conditions across multiple laboratory visits. The picture recognition task served as a basic attention check, but it did not adequately verify task compliance. Participants may not have implemented the intended regulation strategies as instructed. Future research should incorporate more direct assessments of strategy use and task adherence. Moreover, the sample size was relatively moderate. Although each group initially included 32 participants, fewer than 30 participants per group were retained in the fNIRS analyses. This may have limited the statistical power and robustness of the findings, and may partly explain why the fNIRS effects did not survive ROI correction or were no longer significant after covariate adjustment.

## Figures and Tables

**Table 1:** Sample characteristics and group differences between trained and untrained participants.

	Trained Group (*n* = 32)	Untrained Group (*n* = 32)	*p* Values
Age	20.34 ± 0.35	20.84 ± 0.39	0.348
BMI	21.72 ± 0.42	21.35 ± 0.50	0.573
Resting HR (bpm)	71.69 ± 1.98	77.09 ± 1.77	0.046 *
Years of education	13.94 ± 0.32	14.19 ± 0.33	0.593
Beck depression inventory	2.66 ± 0.60	2.81 ± 0.69	
Total mood disturbance	92.53 ± 2.80	92.44 ± 4.28	0.985
State-trait anxiety inventory—Trait	36.44 ± 1.40	35.94 ± 1.67	0.819
**IPAQ**			
MET scores	3037.50 ± 357.73	882.52 ± 82.29	<0.001 ***
Sedentary time	236.78 ± 25.00	288.06 ± 21.28	0.124
**PANAS**			
Positive affect	29.06 ± 1.29	30.78 ± 1.18	0.329
Negative affect	12.81 ± 0.66	13.75 ± 1.26	0.028 *
**Emotion regulation Questionnaire**			
Reappraisal	32.91 ± 0.80	33.69 ± 0.69	0.463
Suppression	16.88 ± 0.96	14.81 ± 1.11	0.164

BMI = body mass index; Total Mood Disturbance = total score on the profile of mood states; MET = metabolic equivalent of task; PANAS = Positive and Negative Affect Schedule. Data are presented as mean ± SE. Asterisks indicate significant between-group differences, *p* < 0.05 *, *p* < 0.001 ***.

## Data Availability

The data presented in this study are available on reasonable request from the corresponding author. The datasets generated during the current study are not publicly available due to ethical and privacy considerations associated with neural activity and emotional data.

## Appendix A

**Table A1:** Valence and Arousal of picture sets used in the emotion regulation task.

	IAPS Parameters		Raters’ Evaluations	
	Arousal	Valence	Arousal	Valence
WNeu	3.44 ± 0.15	5.08 ± 0.04	3.88 ± 0.13	4.80 ± 0.10
WNeg	5.61 ± 0.19	2.45 ± 0.10	6.13 ± 0.18	2.72 ± 0.15
CR	5.46 ± 0.13	2.52 ± 0.10	5.90 ± 0.15	3.04 ± 0.13
ES	5.58 ± 0.15	2.76 ± 0.11	5.71 ± 0.14	3.14 ± 0.13

Values represent mean ± standard error (M ± SE). Arousal was rated on a 9-point scale (1 = very calm, 9 = very excited), and valence on a 9-point scale (1 = very unpleasant, 9 = very pleasant).

**Table A2:** Valence and Arousal of picture sets used in the emotion recall task.

		Arousal		Valence	
		*M* ± *SE*	*p*	*M* ± *SE*	*p*
Neutral	New (*n* = 6)	4.51 ± 0.47	0.354	5.26 ± 0.20	0.321
	Old (*n* = 6)	3.93 ± 0.36		4.89 ± 0.29	
Negative	New (*n* = 18)	5.69 ± 0.16	0.214	3.31 ± 0.14	0.145
	Old (*n* = 18)	6.06 ± 0.24		2.92 ± 0.22	

**Table A3:** Behavioral results of emotion regulation task before and after controlling for baseline PANAS negative affect.

Outcome	Effect	Before Covariate Adjustment	*p*	$\eta_{p}^{2}$	After Covariate Adjustment	*p*	$\eta_{p}^{2}$
Emotional elicitation	Trained	*F*(1, 62) = 5.901	0.018	0.087	*F*(1, 61) = 5.658	0.021	0.085
	R	*F*(1, 62) = 194.006	<0.001	0.758	*F*(1, 61) = 27.958	<0.001	0.314
	Trained × R	*F*(1, 62) = 8.349	0.005	0.119	*F*(1, 61) = 8.122	0.006	0.118
Cognitive reappraisal	Trained	*F*(1, 62) = 4.751	0.033	0.071	*F*(1, 61) = 4.468	0.039	0.068
	R	*F*(1, 62) = 97.851	<0.001	0.612	*F*(1, 61) = 17.994	<0.001	0.228
	Trained × R	*F*(1, 62) = 7.297	0.009	0.105	*F*(1, 61) = 7.366	0.009	0.108
Expressive suppression	Trained	*F*(1, 62) = 5.406	0.023	0.080	*F*(1, 61) = 5.036	0.028	0.076
	R	*F*(1, 62) = 33.707	<0.001	0.352	*F*(1, 61) = 13.995	<0.001	0.187
	Trained × R	*F*(1, 62) = 1.307	0.257	0.021	*F*(1, 61) = 1.653	0.203	0.026

**Table A4:** fNIRS results before and after controlling for baseline PANAS negative affect.

Outcome	Effect	Before Covariate Adjustment	*p*	$\eta_{p}^{2}$	After Covariate Adjustment	*p*	$\eta_{p}^{2}$
Emotion elicitation: rVLPFC	Trained	*F*(1, 56) = 4.492	0.039	0.074	*F*(1, 55) = 4.207	0.045	0.071
	R	*F*(1, 56) = 0.137	0.713	0.002	*F*(1, 55) = 0.432	0.514	0.008
	Trained × R	*F*(1, 56) = 2.399	0.127	0.041	*F*(1, 55) = 2.262	0.138	0.039
Cognitive reappraisal: rVLPFC	Trained	*F*(1, 56) = 5.112	0.028	0.084	*F*(1, 55) = 4.815	0.032	0.080
	R	*F*(1, 56) = 0.780	0.381	0.014	*F*(1, 55) = 0.000	0.994	0.000
	Trained × R	*F*(1, 56) = 1.262	0.266	0.022	*F*(1, 55) = 1.188	0.280	0.021
Cognitive reappraisal: rDLPFC	Trained	*F*(1, 56) = 0.715	0.401	0.013	*F*(1, 55) = 0.764	0.386	0.014
	R	*F*(1, 56) = 5.050	0.029	0.083	*F*(1, 55) = 1.099	0.299	0.020
	Trained × R	*F*(1, 56) = 1.396	0.242	0.024	*F*(1, 55) = 1.343	0.252	0.024
Expressive suppression: rDLPFC	Trained	*F*(1, 56) = 0.274	0.603	0.005	*F*(1, 55) = 0.248	0.621	0.004
	R	*F*(1, 56) = 1.971	0.166	0.034	*F*(1, 55) = 2.273	0.137	0.040
	Trained × R	*F*(1, 56) = 4.259	0.044	0.071	*F*(1, 55) = 4.505	0.038	0.076
